# 1333. Prognostic Factors Associated with Complications of Acute Hematogenous Osteomyelitis in Children

**DOI:** 10.1093/ofid/ofac492.1163

**Published:** 2022-12-15

**Authors:** Adriana Sarmiento Clemente, Jonathon C McNeil, Kristina G Hulten, Jesus G Vallejo, Sheldon L Kaplan

**Affiliations:** Baylor College of Medicine, Houston, Texas; Baylor College of Medicine, Houston, Texas; Baylor College of Medicine, Houston, Texas; Baylor College of Medicine, Houston, Texas; Baylor College of Medicine, Houston, Texas

## Abstract

**Background:**

The clinical presentation and management of acute hematogenous osteomyelitis (AHO) in children can vary significantly. Scores to predict acute complications utilize prolonged fever after antibiotics, bone abscess, suppurative arthritis, disseminated infection and delayed source control. By contrast, elevated CRP after 48-96 hours of therapy, disseminated disease and bone debridement are predictive of chronic complications. Such scoring systems have undergone limited validation. We examined factors associated with acute and chronic AHO complications at our center to identify other variables that may enhance published scores.

**Methods:**

A retrospective chart review was conducted of all children 6 mo-18 years with AHO and with acute symptoms of < 14 days at Texas Children’s Hospital from January 2012 through December 2020. An acute complicated course was defined as treatment failure within 6 weeks of starting antibiotics, > 2 bone debridements, prolonged admission ( >14 days) and acute avascular necrosis. Chronic complications included growth arrest or limb leg discrepancy, pathologic fracture, avascular necrosis, chronic osteomyelitis or frozen joint. Statistical analysis was completed using STATA 17.

**Results:**

418 patients met the inclusion criteria. 106 (25.4%) had an acute complicated course. 51 (13.5%) of 377 followed had a chronic complication. Clinical factors associated with acute and chronic complications were very similar. Factors associated with either an acute complicated course (Figure 1) or chronic orthopedic complications (Figure 2) included: older age, tibia involvement, infection due to *S. aureus*/MRSA, presence of multifocal or disseminated infection, DVT, fever > 48 hours of antibiotic therapy, admission laboratory values including higher absolute neutrophil count (ANC) and higher CRP, ICU admission, associated suppurative arthritis, bacteremia, bone abscess, surgical debridement and delayed source control.

**Figure:**
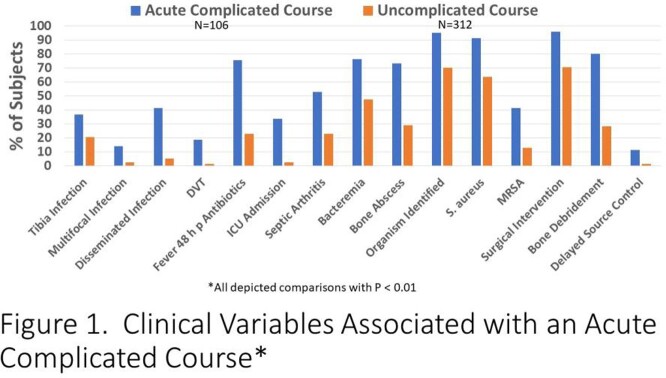

**Figure:**
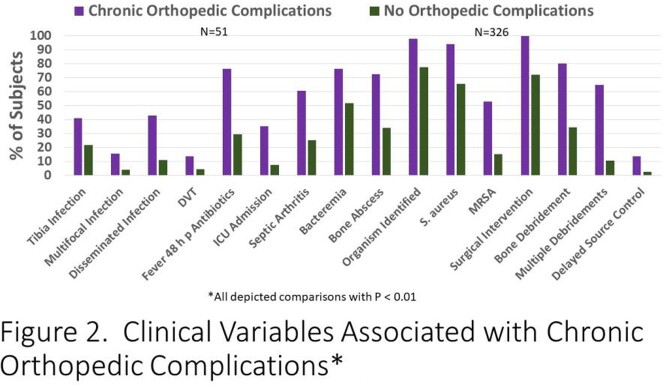

**Conclusion:**

In children with AHO, risk factors for acute and chronic complications are highly similar. Older age, tibia involvement, MRSA, higher ANC and bacteremia were linked to complications in our population and will be assessed in current clinical scoring systems which may help guide management.

**Disclosures:**

**Jonathon C. McNeil, MD**, Agency for Healthcare Research and Quality: Grant/Research Support|Allergan: Provided reagents for unrelated research|Nabriva: Site investigator for multicenter clinical trial **Kristina G. Hulten, PhD**, Me-med: Grant/Research Support|Pfizer: Grant/Research Support **Sheldon L. Kaplan, MD**, MeMed: Grant/Research Support|Pfizer: Grant/Research Support.

